# Prevalence, correlates, and reasons for substance use among adolescents aged 10–17 in Ghana: a cross-sectional convergent parallel mixed-method study

**DOI:** 10.1186/s13011-024-00600-2

**Published:** 2024-02-29

**Authors:** Sylvester Kyei-Gyamfi, Frank Kyei-Arthur, Nurudeen Alhassan, Martin Wiredu Agyekum, Prince Boamah Abrah, Nuworza Kugbey

**Affiliations:** 1 Department of Children, Ministry of Gender, Children and Social Protection, Accra, Ghana; 2 Department of Environment and Public Health, University of Environment and Sustainable Development, Somanya, Ghana; 3 African Institute for Development Policy, Lilongwe, Malawi; 4 Institute for Educational Research and Innovation Studies, University of Education, Winneba, Ghana; 5 Department of Social Welfare, Ministry of Gender, Children and Social Protection, Accra, Ghana; 6 Department of General Studies, University of Environment and Sustainable Development, Somanya, Ghana

**Keywords:** Substance use, Risk factors, Protective factors, Adolescents, Ghana

## Abstract

**Background:**

Substance use among adolescents poses significant risks to their health, wellbeing, and development, particularly in low- and middle-income countries, including Ghana. However, little is known about the outlets and reasons for substance use among Ghanaian adolescents. This study examined the prevalence, correlates, reasons for substance use, and outlets of these substances among adolescents aged 10–17 in Ghana.

**Methods:**

Data were obtained from the Department of Children, Ministry of Gender, Children, and Social Protection, Ghana, which employed a cross-sectional convergent parallel mixed-methods technique to collect quantitative and qualitative data from children aged 8–17, parents or legal guardians and officials of state institutions responsible for the promotion and protection of children’s rights and wellbeing. Overall, 4144 adolescents aged 10–17 were interviewed for the quantitative data, while 92 adolescents participated in 10 focus group discussions. Descriptive statistics, Pearson’s chi-square test, and multivariable binary logistic regression were used to analyse the quantitative data, while the qualitative data was analysed thematically.

**Results:**

The prevalence of substance use was 12.3%. Regarding the types of substance use, alcohol (56.9%) and cigarettes (26.4%) were the most common substances. Being a male and currently working are significant risk factors, whereas being aged 10–13, and residing in the Middle- and Northern-ecological belts of Ghana are significant protective factors of substance use. Peers, household members who use substances, drug stores, and drug peddlers are the major outlets. The reasons for substance use were fun, substance as an aphrodisiac, boosting self-confidence, dealing with anxiety, and improved social status.

**Conclusions:**

There is a relatively high substance use among adolescents in Ghana, and this calls for a multi-sectoral approach to addressing substance use by providing risk-behaviour counselling, parental control, and effective implementation of substance use laws and regulations.

**Supplementary Information:**

The online version contains supplementary material available at 10.1186/s13011-024-00600-2.

## Background

Adolescence is a developmental phase associated with a greater risk of experimenting and using substances such as alcohol, cannabis and tobacco [1]. Substance use among adolescents is of major public health concern because of the short-and long-term effects on their health and safety as well as the broader negative social consequences [2–4]. Specifically, substance use is associated with an increased risk of road traffic accidents, violence, sexual risk-taking (such as unprotected sex), mental health disorders (including learning disorders) and suicide. While substance use among adolescents is not new in Ghana, there is evidence of the rising prevalence of some substances and the use of ‘new substances’ (such as tramadol), which have greater intoxicating effects [5]. According to Kyei-Gyamfi et al. [5], about 7% of children aged 8–17 in Ghana are lifetime users of alcohol.

In Ghana, multiple laws forbid and govern the use and sale of substances to individuals under 18. For instance, the sale of tobacco products to individuals under the age of 18 is regulated by the Tobacco Control Regulations 2016 (L. I. 2247) [6] and the Public Health Act, 2012 (Act 851) [7]. Specifically, the Public Health Act, 2012 (Act 851) forbids smoking tobacco products in public places and advertisements on tobacco products. In Ghana, anti-smoking campaigns, such as the SKY Girls campaign, employed diverse channels, including school and community activities, films, and social media, to dissuade adolescents from smoking [8, 9]. The Food and Drugs Authority guidelines for the advertisement of foods [10] stipulate that advertisements for alcoholic beverages should not appeal to or target individuals under 18. Consequently, the Food and Drugs Authority is responsible for examining and authorising all advertisements related to alcoholic beverages. In addition, alcoholic beverage companies are prohibited from selling or providing their products as prizes for sponsorship programmes at educational institutions.

Also, the Liquor Licensing Act 1970 (Act 331) [11] regulates the sale of alcoholic beverages to individuals under 18. Act 331 also stipulates that individuals under 18 should not be permitted to enter or be found in any premises where alcoholic beverages are sold. Furthermore, the Narcotic Drugs (Control, enforcement and Sanctions) Act, 1990 (P.N.D.C.L. 236) [12] forbids the utilisation of narcotic drugs by any individual without legal authorization, including children.

While a body of research exists on substance use in Ghana, it is essential to acknowledge some limitations associated with these studies. First, these studies have mainly used a type of substance to measure substance use (e.g., alcohol use, tobacco use, and shisha use) [2, 5, 13–19]. For instance, Kugbey’s [2] study measured substance use among adolescents using alcohol use, amphetamine use, and marijuana use. Similarly, Asante and Nefale [18] estimated substance use using alcohol use, cigarette use, marijuana use, glue, heroin, and amphetamine. To the best of our knowledge, no study in Ghana has used varieties of substance use as a composite variable to measure substance use. Second, most studies have focused on in-school adolescents [2, 13–16]. Third, few studies have interrogated the various sources and outlets where adolescents procure substances [5]. Therefore, this study examined the prevalence, correlates and reasons for substance use as well as the outlets where such substances are procured among adolescents aged 10–17 in Ghana.

## Methods

### Data and sample

The study employed secondary data as the primary source of information. The data was acquired from the Department of Children within the Ministry of Gender, Children, and Social Protection in Ghana, which employed a cross-sectional convergent parallel mixed-method technique to collect quantitative and qualitative data from children aged 8–17, parents or legal guardians and officials of state institutions responsible for the promotion and protection of children rights and wellbeing. A convergent parallel mixed-method technique enables the simultaneous gathering and examination of quantitative and qualitative data [20]. The secondary data cover several topics, including children’s rights, substance use, employment, and sexual and reproductive health. This study focused on substance use.

**Fig. 1:**
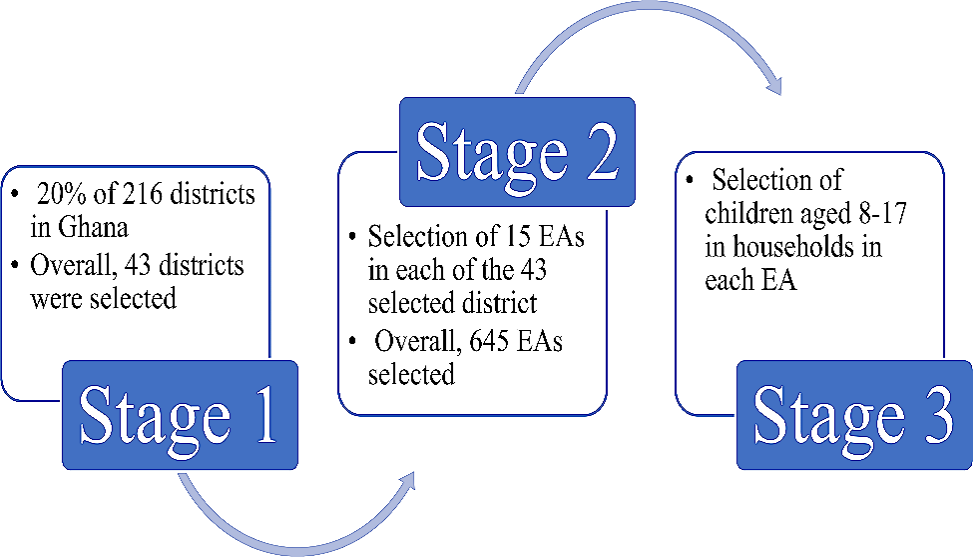
Multi-stage sampling procedure for the quantitative data collection

The quantitative data in this study was obtained using a multi-stage sampling procedure to select respondents. In 2018, a sample of 20% of the total 216 districts in Ghana was chosen, with the selection criteria focusing on child welfare issues, such as child rights and child protection. As a consequence, a total of 43 districts were chosen. Furthermore, 645 enumeration areas were selected by choosing 15 enumeration areas in each of the selected 43 districts. Moreover, the study involved the selection of children between the ages of 8 and 17 residing in households within each enumeration area. In each household, it was ensured that just one child between the ages of 8 and 17 was selected for the interview. However, this study focused on adolescents aged 10–17. Overall, 4144 adolescents aged 10–17 were interviewed for the study. Figure 1 is an organisational flow of the multi-stage sampling procedure for the quantitative data collection. The inclusion criteria for children to participate in the study were: they must be aged 8–17, be a member of eligible households in selected EAs, must consent and be willing to participate, and parents/legal guardians must consent for them to participate in the study.

In order to gather qualitative data, ten focus group discussions (FGDs) were carried out with adolescents aged 10 to 17 years at locations convenient for them. Each FGD consisted of 8–10 participants, encompassing male and female adolescents across various age groups. Overall, 92 adolescents aged 10–17 participated in the ten FGDs. Topics covered in the FGDs included the types of substances adolescents use, where adolescents get substances to use, and the reasons for the use of substances.

The study was approved by the National Child Protection Committee of the Department of Children of the Ministry of Gender, Children, and Social Protection. Adolescents gave their written informed consent before trained research assistants interviewed them. Also, research assistants received written informed consent from parents or legal guardians of eligible adolescents before interviewing them. Experienced research assistants proficient in mixed-method data collection were enlisted and underwent a comprehensive training session on the data collection tools, as well as the objectives and significance of the study. Further information regarding the sampling process might be obtained in prior studies [21].

### Study variables

#### Dependent variable

Substance use was the dependent variable for this study. It was a composite variable computed using two questions, “Have you ever taken alcohol?” and “Have you ever taken drugs?”. Respondents who responded “Yes” to either or both questions were classified as engaging in substance use.

#### Independent variables

The independent variables were sex (Male and Female), age (10–13 and 14–17), education (Less than Junior High School (JHS), JHS, and Senior High School (SHS) and higher), marital status (married and not married), religion (Christianity, Islam, and Other), and currently doing any paid work (Yes and No). The region of residence of respondents was recoded into three (3) ecological belts: Coastal ecological belt (Western, Central, Greater Accra, and Volta regions), Middle ecological belt (Eastern, Ashanti and Brong Ahafo regions), and Northern (Northern, Upper East and Upper West regions) ecological belt.

### Statistical analyses

The Statistical Package for the Social Sciences (SPSS) version 26 was used to perform the statistical analyses for the quantitative data. Descriptive statistics was used to describe the socio-demographic characteristics of respondents and the types of substances respondents use. Pearson’s chi-square test was used to examine the association between substance use and socio-demographic characteristics of respondents. A multivariable binary logistic regression was performed to examine the correlates of substance use. All variables in the quantitative data were determined to be statistically significant at p-value ≤ 0.05.

QSR NVivo version 10 software was used to analyse the qualitative data thematically. The researchers examined all transcripts to gain insights into respondents’ viewpoints regarding substance use among adolescents. Subsequently, the transcripts were thoroughly examined, and utterances pertaining to the substance usage of the adolescents were systematically categorised using codes. Sub-themes were identified from the identification and grouping of similar codes found within the transcripts. Moreover, the process involved clustering comparable sub-themes to generate overarching themes.

## Results

### Socio-demographic characteristics of respondents

The socio-demographic characteristics of respondents are displayed in Table 1. A little more than half of the respondents (50.7%) were males, while most were Christians (76.4%). Most respondents were unmarried (98.9%) and currently not engaged in any paid work (95.2%). Among those currently working, a higher proportion were males (68.3%) than females (31.7%) (See Table S1 in the supplementary material).

**Table 1:** Socio-demographic characteristics and substance use by socio-demographic characteristics

Variables		Substance use		*P*-values
	Frequency (%)	Yes (%)	No (%)	
*Sex*				≤ 0.001
Male	2099 (50.7)	331 (15.8)	1768 (84.2)	
Female	2045 (49.3)	180 (8.8)	1865 (91.2)	
*Age*				≤ 0.001
10–13	1947 (47.0)	114 (5.9)	1833 (94.1)	
14–17	2197 (53.0)	397 (18.1)	1800 (81.9)	
*Religion*				0.091
Christianity	3167 (76.4)	388 (12.3)	2779 (87.7)	
Islam	840 (20.3)	98 (11.7)	742 (88.3)	
Other	137 (3.3)	25 (18.2)	112 (81.8)	
*Marital status*				0.264
Married	45 (1.1)	8 (17.8)	37 (82.2)	
Not married	4099 (98.9)	503 (12.3)	3596 (87.7)	
*Education*				≤ 0.001
Less than JHS	1263 (30.5)	72 (5.7)	1191 (94.3)	
JHS	1584 (38.2)	190 (12.0)	1394 (88.0)	
SHS and higher	1297 (31.3)	249 (19.2)	1048 (80.8)	
*Ecological belts*				≤ 0.001
Coastal	1563 (37.7)	260 (16.6)	1303 (83.4)	
Middle	1642 (39.6)	151 (9.2)	1491 (90.8)	
Northern	939 (22.7)	100 (10.6)	839 (89.4)	
*Currently doing any paid work*				
Yes	199 (4.8)	56 (28.1)	143 (71.9)	≤ 0.001
No	3945 (95.2)	455 (11.5)	3490 (88.5)	
**Total**	**4144 (100.0)**	**511 (12.3)**	**3633 (87.7)**	

More than half of respondents (53.0) were aged 15–17. Also, 3 out of 10 respondents (30.5%) had attained less than JHS education, and about two-fifths (39.6%) resided in the Middle ecological belt.

### Prevalence of substance use

The prevalence of substance use was 12.3% (95% CI = 11.34 − 13.37%) (Table 1). In terms of sex, a greater proportion of males (15.8%) engaged in substance use than females (8.8%, *p* ≤ 0.001). More respondents aged 15–17 (18.1%) engaged in substance use than those aged 10–13 (5.9%, *p* ≤ 0.001). It can be observed that respondents’ education was positively associated with the prevalence of substance use (*p* ≤ 0.001). Most respondents with SHS and higher education (19.2%) engaged in substance use, followed by those with JHS (12.0%) and less than JHS (5.7%) education. Also, most respondents who resided in the Coastal ecological belt (16.6%, *p* ≤ 0.001) and those who are currently doing paid work (28.1%, *p* ≤ 0.001) engaged in substance use.

Regarding the type of substance respondents use, alcohol (56.9%) and cigarettes (26.4%) were the most common substances (Table 2). About 7% of respondents (6.5%) used tramadol, and 4.8% used marijuana. Other substances respondents used include codeine (1.7%), cocaine (1.5%), shisha (1.2%), and heroin (1.0%).

**Table 2:** Type of substance respondents use by sex and age of adolescents

Substance respondents use	Frequency (%)	Sex		Age	
		Male	Female	10–13	14–17
		Frequency (%)	Frequency (%)	Frequency (%)	Frequency (%)
Alcohol	333 (56.9)	181 (54.4)	152 (45.6)	81 (24.3)	252 (75.7)
Cigarette	154 (26.4)	135 (87.7)	19 (12.3)	18 (11.7)	136 (88.3)
Tramadol	38 (6.5)	33 (86.8)	5 (13.2)	4 (10.5)	34 (89.5)
Marijuana	28 (4.8)	23 (82.1)	5 (17.9)	4 (14.3)	24 (85.7)
Codeine	10 (1.7)	9 (90.0)	1 (10.0)	3 (30.0)	7 (70.0)
Cocaine (crack, rock)	9 (1.5)	7 (77.8)	2 (22.2)	4 (44.4)	5 (55.6)
Shisha	7 (1.2)	6 (85.7)	1 (14.3)	3 (42.9)	4 (57.1)
Heroin (smack, horse)	6 (1.0)	5 (83.3)	1 (16.7)	4 (66.7)	2 (33.3)
**Total**	585* (100.0)	399* (100.0)	186* (100.0)	121* (100.0)	464* (100.0)

* Multiple responses

Generally, more male adolescents used all types of substances than female adolescents. For instance, of those adolescents who used codeine, 90% were male, and of those who used tramadol, 86.8% were male. Similarly, older adolescents (14–17 years) generally used all types of substances than younger adolescents (10–13 years), except heroin. For instance, about 9 out of 10 older adolescents (89.5%) used tramadol, while 88.3% used cigarettes. However, more younger adolescents (66.7%) used heroin than older adolescents (33.3%).

### Correlates of substance use

From Table 3, males (AOR = 2.117, 95% C.I. = 1.731–2.589, *p* ≤ 0.001) and respondents who are currently working (AOR = 1.821, 95% C.I. = 1.295–2.560, *p* = 0.001) were more likely to engage in substance use. However, respondents aged 10–13 (AOR = 0.333, 95% C.I. = 0.232–0.478, *p* ≤ 0.001) and residing in the Middle (AOR = 0.510, 95% C.I. = 0.409–0.636, *p* ≤ 0.001) and Northern (AOR = 0.597, 95% C.I. = 0.451–0.790, *p* ≤ 0.001) ecological belts were less likely to engage in substance use.

**Table 3:** Correlates of substance use among adolescents

	AOR	95% C.I. for AOR		P-value
		Lower	Upper	
*Sex*				
Male	2.117	1.731	2.589	≤ 0.001
Female (RC)				
*Age*				
10–13	0.333	0.232	0.478	≤ 0.001
14–17 (RC)				
*Religion*				
Christianity	0.755	0.473	1.205	0.239
Islam	0.782	0.470	1.300	0.343
Other (RC)				
*Marital status*				
Married	1.408	0.632	3.134	0.403
Not married (RC)				
*Education*				
Less than JHS (RC)				
JHS	1.053	0.710	1.561	0.798
SHS and higher	1.283	0.813	2.023	0.284
*Ecological belts*				
Coastal (RC)				
Middle	0.510	0.409	0.636	≤ 0.001
Northern	0.597	0.451	0.790	≤ 0.001
*Currently doing any paid work*				
Yes	1.821	1.295	2.560	0.001
No (RC)				

## Where respondents get substance

During the FGD with respondents, issues were discussed regarding where children get their substance. Four themes emerged: (a) supplies from their peers, (b) household members who also use substances, (c) purchasing from drug stores, and (d) purchasing from drug peddlers.

### (A) supplies from peers

Respondents explained that friends with connections can provide access to illicit substances. They explained that most adolescents who engage in substance use are extremely cautious when searching for their drug of choice, as they are aware that the simplest act of irresponsibility will get them in trouble. As a result, they rely on their peers who also engage in substance use since they can trust them.

Most drug-using young people only associate with peers who also use drugs. Through this, they can establish a network, gain each other’s trust, and obtain supplies, as suppliers find it convenient to give substances to trustworthy individuals. Once a member obtains supplies, they may distribute them within their respective circles. (FGD 1)

### (B) household members who use substances

Household members who engage in substance use also serve as suppliers of substances. It was found that some adolescents obtain substances from their siblings, uncles, and other household members who also use substances. One participant explained this phenomenon:

”My first taste of whisky came from my older brother’s room. Since I frequently observe him drinking before meals, I decided to try it one day, and it has since become my primary source of alcohol. Some of my smoking acquaintances obtain their supplies from their brothers, too. (FGD 2)”.

### (C) purchasing from drug stores

The FGDs with adolescents revealed that some adolescents acquire tramadol from small medicinal retail outlets known as ‘drug stores’. Adolescents explained that acquiring such medications from a pharmacy is risky due to the possibility of being tracked down for drug use. A participant explained:

When customers enter small drugstores in densely populated communities, they are rarely asked what they intend to use the medication for. Not so with pharmacies, which are well-established and staffed by licenced pharmacists. Due to this, some adolescents purchase codeine and tramadol from them to avoid getting caught. (FGD 3)

### (D) purchasing from gangs

Adolescents’ narratives highlighted that they purchase substances from gangs, who often get their supplies from drug peddlers. However, the gangs only sell to individuals they know and can vouch that they may not expose them to the police. Adolescents explained that one must be known to be a reliable client before buying from a gang. These gangs are not at specific locations but operate in areas noted for substance use.

Drug peddlers who occasionally offer marijuana do not sell to adolescents. Typically, an adult member of a gang buys it and distributes it around the groups, usually on the basis that he receives a free supply, while adolescents or underage members provide monies for the purchase of the marijuana. (FGD 4)

### Reasons for substance use

Adolescents narrated varied reasons for using substances. Five themes emerged: (a) substance use is fun, (b) use of substances as an aphrodisiac, (c) boosts confidence to approach the opposite sex, (d) forgetting anxieties, and (e) substance use makes one popular and being perceived as the finest.

### (A) substance use is fun

For some adolescents, substance use is fun. Occasionally, meeting friends, drinking, and smoking add spice to the entertainment of adolescents. One of the adolescents described how he has enjoyed drinking with his neighbourhood friends over the years:

The greatest time of my life is when my friends and I assemble at drinking places [pubs], listen to music, dance to some songs, consume alcohol, smoke, and party. Life is great when unwinding with friends. (FGD 5)

### (B) use of substances as an aphrodisiac

During the FGD, it emerged that the use of tramadol, also known as ‘Tramol’, has become widespread among many adolescents in the country’s urban communities since it serves as an aphrodisiac, which enhances their sexual performance. One adolescent stated:

Boys who use Tramol in my area claim that it allows them to have long-lasting sexual intercourse with their partners whenever they have sex. As a result, many adolescents use Tramol as an aphrodisiac. (FGD 6)

### (C) boosts confidence to approach the opposite sex

Some adolescents believe that when they use substances, it boosts their confidence to approach the opposite sex. They use substances to overcome their shyness to approach the opposite sex. Below is the narrative of a respondent:

Some of the boys drink alcohol or even smoke marijuana because they may lack the confidence to approach a girl they are interested in. However, they firmly believe that by using drugs, they will become high and be better positioned to accomplish their goal of flirting with the girls. (FGD 7)

### (D) forgetting anxieties

The FGD with adolescents revealed that some adolescents use substances to help them forget about their anxieties about the lack of employment opportunities, educational opportunities, and other family issues. One participant explained why he has been drinking:

I finished Polytechnic and have been home for two years without a job. Most of my peers are in similar situations, so when we get together, those of us with money purchase drinks and split them amongst ourselves so we can commiserate and console ourselves about our jobless situations. (FGD 8)

### (E) substance use makes one popular and being perceived as the finest

Adolescents revealed that some adolescents have the misconception that engaging in substance use makes them popular and perceived as the ‘finest’ [best] in their peer group. The following were the sentiments expressed:

Most children who smoke cigarettes and marijuana believe they become popular with their peers and are highly regarded as the finest guys in their group when they smoke. Many boys and girls of my age group are influenced to indulge in substance use due to the widespread prevalence of these misconceptions. (FGD 9)

## Discussion

Although there have been many studies conducted on substance use in Ghana, only a limited number of these studies have utilised multiple types of substances to assess substance use. Moreover, most of these studies have concentrated on adolescents attending school. At the same time, only a limited number of studies have examined the diverse channels and locations through which adolescents obtain substances. To close this disparity in knowledge, this study examined the prevalence, correlates and reasons for substance use as well as the outlets where such substances are procured among adolescents aged 10–17 in Ghana. The findings of this study would contribute to the literature on substance use, and it would help to inform policymakers in providing programmatic responses to address substance use among adolescents, which has long-term health implications for their lives.

We found that the prevalence of substance use among adolescents was 12.3%. The prevalence of substance use in this study is higher than that of 6.6% found in 7.2% in Uganda [22] and 11.3% in sub-Saharan Africa [2]. In contrast, the prevalence of substance use in this study is lower than 16.0% in India [23], 17.1% in Southern Brazil [24], 32.9% in Nigeria [25], and 48% in South Africa [26]. A study in Northern Tanzania reported a lifetime and current prevalence of substance use of 19.7% and 12.8%, respectively [27]. The variation in the prevalence of this study and other studies could be attributed to the sample size of adolescents, socio-cultural factors, demographic characteristics, age of adolescents and the types of substance use that were considered in each study. For instance, Mavura et al. [27] considered alcohol, cigarette smoking, marijuana, khat, and recreational drugs (cocaine, heroin) in their study, while in this study, we considered alcohol, smoking and drugs (Marijuana, heroin, cocaine, codeine and tramadol). In addition, the differences in the age of adolescents could account for the variations in the prevalence of substance use among adolescents. In this study, we considered adolescents aged 10–17. However, Kugbey’s [2] study participants were aged 11–18, while Mmereki et al.’s [26] study participants were aged 13–21.

The results of this study show that alcohol was the most common substance used among adolescents, followed by smoking (cigarettes), tramadol, marijuana, codeine, cocaine, shisha and heroine. The findings of this study are similar to a study by Mavura et al. [27], who reported alcohol as the common substance use by adolescents in Tanzania. Similar findings were found by Anyanwu et al. [25] in Nigeria, Birhanu et al. [28] in Northwest Ethiopia, and Olawole-Isaac et al. [29] in Sub-Saharan Africa. The probable reason for the high prevalence of alcohol use may be attributed to the visibility and advertisement of alcohol in Ghana, which may entice adolescents to drink. Akesse-Brempong and Cudjoe [30] argued that there is a pervasive and robust advertising campaign promoting the consumption of alcoholic beverages in Ghana, which has the potential to influence adolescents to consume alcohol. Aside from the advertisement, there are more drinking spots where adolescents can easily access any alcoholic beverage. Hormenu et al. [14] reported that in Ghana, about 42.3% of adolescents have ever consumed alcohol. It is, therefore, not surprising that alcohol was the major substance used by adolescents in this study. In addition, studies have identified smoking cigarettes as the second substance adolescents use [25, 27, 28]. In contrast, Srivastava et al. [23] found the use of tobacco to be higher in India than alcohol.

The findings of the study show that males were more likely to engage in substance use than females, similar to other studies [2, 5, 28, 31]. The higher use of substances among males may be attributed to gender roles, peer influence and sensation-seeking behaviour, which sometimes forces males to use substances to enable them to behave as they desire [28]. Iwamoto et al. [32] reported that substance use, such as alcohol and drugs, shows masculinity, whereas men who do not take alcohol or drugs are considered weak. Men conform to these masculine norms or beliefs, such as “playboy” and “risk-taking and self-reliance”, which increase their risk of substance use. In contrast, engaging in substance use among females is sometimes seen as shameful, and society frowns on it [2]. Due to this, there could be a situation of underreporting of substance use among adolescent females.

The study results show that adolescents aged 10–13 years were less likely to engage in substance use than those aged 14–17 years. The finding of this study is similar to other studies that reported substance use practice among older adolescents than younger adolescents [25, 26]. The probable reason could be that as adolescents grow, they begin to live independent lives by making their own decisions, and this sometimes leads them to engage in unhealthy lifestyles such as drinking alcohol and taking drugs. Older adolescents become susceptible to experimentation with different things, such as drugs and alcohol. Sometimes, this is done out of curiosity or peer pressure from friends as they age [25, 33]. However, older adolescents may lack the knowledge and consequences of using these substances, and their continuous use may lead to addiction.

In addition, we found that adolescents who were currently working were more likely to engage in substance use than those who were not working. Adolescents who are working may have the financial resources to purchase substances to use than those who are not working. This finding is similar to previous studies, which found that respondents who were working as vulnerable groups that engaged in substance use [23, 34, 35]. However, this finding is contrary to Masferrer et al. [36] study, which found substance use is more likely among respondents who are not working.

Furthermore, adolescents residing in the Coastal ecological belt are more likely to engage in substance use than those living in the Middle and Northern ecological belts. Previous studies have found the use of alcohol among persons residing in Coastal areas in Ghana [37]. Also, the use of tramadol has been documented to be more prevalent in the Greater Accra, Volta, and Western regions, which are found in the Coastal ecological belt [38]. Kyei-Gyamfi and Kyei-Arthur’s [19] study on substance smoking in Ghana found cigarette smoking to be more prevalent in the Coastal ecological belt than in the Middle and Northern belts of Ghana. These factors may explain why adolescents living in the Coastal ecological belt are more likely to use substances than those in the Middle and Northern ecological belts.

Consistent with other studies [39, 40], the findings of this study revealed various reasons for substance use, such as for fun, as an aphrodisiac, to boost confidence to approach the opposite sex, forgetting anxieties, making one popular and being perceived as the finest. The probable reason for this could be that most adolescents are sometimes shy of approaching the opposite sex. Therefore, they use substances to boost their confidence to approach them. In addition, due to youthful exuberances, adolescents are involved in risky sexual behaviour and, therefore, use various substances as aphrodisiacs to please their partners during sex [41]. In Ghana, Attila et al. [42] reported that substance use among adolescents is due to curiosity, which is similar to the findings of this study.

The study found that adolescents obtained their substances from peers, household members who engage in substance use, drug stress and gangs. Similar findings of this study have been reported by other studies [43, 44]. For instance, Lopez-Mayan & Nicodemo [43] reported that peers significantly influence adolescent substance use in Spain. These adolescents get the substances from their peers in schools, thereby impacting their use. Schuler et al.’s [44] study in Southern California reported that adolescents acquire the substance they use from their friends and family members. Similarly, Srivastava et al.’s [23] study in India found that the probability of an adolescent engaging in substance use is heightened when they have a family member who also engages in substance use. Thus, peers and family members who use substances may influence adolescents to also use substances.

### Limitations

There are some limitations to this study. First, substance use was defined as the lifetime use of a substance, and it was measured by a single question, which was not a robust measure of substance use. Second, children were asked if they had ever used substances. Since substance use is regarded as a deviant behaviour and unlawful, children might fail to disclose their use. Third, there may also be recall bias because children must recollect when they used a substance, which may lead to under reporting of their experiences. Fourth, because this is a cross-sectional study, we are unable to establish causal links between the dependent and independent variables. Despite these limitations, the data’s national representativeness would allow policymakers and researchers to tackle substance use among adolescents across the country.

## Conclusions

This study revealed a relatively high prevalence of substance use (12.3%) among adolescents, and alcohol and cigarettes were the main substances used by adolescents. Adolescents obtain the substances they consume from their peers and household members who are substance users, as well as from drug stores and drug peddlers. The study highlighted adolescent’s age, sex, ecological zone of residence and working status as significant correlates of substance use.

Furthermore, it emerged that adolescents use substances because they want to boost their self-confidence to approach the opposite sex, forget their anxieties, and it served as a form of aphrodisiac. Other adolescents use substances since they perceive them as fun, and the use of substances makes their peers perceive them as famous.

Though the study found that only a little over one-tenth of adolescents (12.3%) used substances, substance use is detrimental to the health and wellbeing of adolescents. Consequently, there is a need for muti-sectoral collaborations between institutions mandated to enhance the wellbeing of adolescents and implement substance use laws and regulations, such as the Narcotic Control Authority, the Ministry of Gender, Children, and Social Protection, and other child protection partners, to help reduce adolescent substance use. Also, there is a need to provide risk-behaviour counselling to adolescents and to strengthen parent control to help curb adolescent substance use in Ghana.

### Electronic supplementary material

Below is the link to the electronic supplementary material.

Working status of adolescents by sex.

## Data Availability

The raw data for the findings of this study are freely available from the corresponding author upon request.

## Abbreviations

AOR: Adjusted Odds Ratio
C.I.: Confidence Interval
FGDs: Focus Group Discussions
JHS: Junior High School
MoGCSP: Ministry of Gender, Children and Social Protection
RC: Reference Category
SHS: Senior High School
