# Supplementary material for: Prevalence, correlates, and reasons for substance use among adolescents aged 10–17 in Ghana: a cross-sectional convergent parallel mixed-method study
Source: Subst Abuse Treat Prev Policy. 2024 Feb 29;19:17. doi: 10.1186/s13011-024-00600-2 (PMC10905778; doi:10.1186/s13011-024-00600-2)
Supplement: Supplementary file 1 — Working status of adolescents by sex. [file 13011_2024_600_MOESM1_ESM.docx]

**Table S1: Working status of adolescents by sex**

| **Currently doing any paid work** | **Sex of adolescents** | |  |
| --- | --- | --- | --- |
|  | **Male** | **Female** | **Total** |
|  | **Frequency (%)** | **Frequency (%)** | **Frequency (%)** |
| Yes | 136 (68.3) | 63 (31.7) | 199 (100.0) |
| No | 1963 (49.8) | 1982 (50.2) | 3945 (100.0) |
| **Total** | **2099 (50.7)** | **2045 (49.3)** | **4144 (100.0)** |
